# Age-specific association between blood pressure and vascular and non-vascular chronic diseases in 0·5 million adults in China: a prospective cohort study

**DOI:** 10.1016/S2214-109X(18)30217-1

**Published:** 2018-05-14

**Authors:** Ben Lacey, Sarah Lewington, Robert Clarke, Xiang Ling Kong, Yiping Chen, Yu Guo, Ling Yang, Derrick Bennett, Fiona Bragg, Zheng Bian, Shaojie Wang, Hua Zhang, Junshi Chen, Robin G Walters, Rory Collins, Richard Peto, Liming Li, Zhengming Chen, China Kadoorie Biobank collaborative group, Junshi Chen, Zhengming Chen, Robert Clarke, Rory Collins, Yu Guo, Liming Li, Jun Lv, Richard Peto, Robin Walters, Daniel Avery, Derrick Bennett, Ruth Boxall, Fiona Bragg, Yumei Chang, Yiping Chen, Zhengming Chen, Robert Clarke, Huaidong Du, Simon Gilbert, Alex Hacker, Michael Holmes, Andri Iona, Christiana Kartsonaki, Rene Kerosi, Om Kurmi, Sarah Lewington, Garry Lancaster, Kuang Lin, John McDonnell, Iona Millwood, Qunhua Nie, Jayakrishnan Radhakrishnan, Paul Ryder, Sam Sansome, Dan Schmidt, Paul Sherliker, Rajani Sohoni, Becky Stevens, Iain Turnbull, Robin Walters, Jenny Wang, Lin Wang, Neil Wright, Ling Yang, Xiaoming Yang, Zheng Bian, Ge Chen, Yu Guo, Xiao Han, Can Hou, Jun Lv, Pei Pei, Shuzhen Qu, Yunlong Tan, Canqing Yu, Zengchang Pang, Ruqin Gao, Shaojie Wang, Yongmei Liu, Ranran Du, Yajing Zang, Liang Cheng, Xiaocao Tian, Hua Zhang, Silu Lv, Junzheng Wang, Wei Hou, Jiyuan Yin, Ge Jiang, Xue Zhou, Liqiu Yang, Hui He, Bo Yu, Yanjie Li, Huaiyi Mu, Qinai Xu, Meiling Dou, Jiaojiao Ren, Shanqing Wang, Ximin Hu, Hongmei Wang, Jinyan Chen, Yan Fu, Zhenwang Fu, Xiaohuan Wang, Min Weng, Xiangyang Zheng, Yilei Li, Huimei Li, Yanjun Wang, Ming Wu, Jinyi Zhou, Ran Tao, Jie Yang, Chuanming Ni, Jun Zhang, Yihe Hu, Yan Lu, Liangcai Ma, Aiyu Tang, Shuo Zhang, Jianrong Jin, Jingchao Liu, Zhenzhu Tang, Naying Chen, Ying Huang, Mingqiang Li, Jinhuai Meng, Rong Pan, Qilian Jiang, Weiyuan Zhang, Yun Liu, Liuping Wei, Liyuan Zhou, Ningyu Chen, Hairong Guan, Xianping Wu, Ningmei Zhang, Xiaofang Chen, Xuefeng Tang, Guojin Luo, Jianguo Li, Xiaofang Chen, Xunfu Zhong, Jiaqiu Liu, Qiang Sun, Pengfei Ge, Xiaolan Ren, Caixia Dong, Hui Zhang, Enke Mao, Xiaoping Wang, Tao Wang, Xi Zhang, Ding Zhang, Gang Zhou, Shixian Feng, Liang Chang, Lei Fan, Yulian Gao, Tianyou He, Huarong Sun, Pan He, Chen Hu, Qiannan Lv, Xukui Zhang, Min Yu, Ruying Hu, Hao Wang, Yijian Qian, Chunmei Wang, Kaixue Xie, Lingli Chen, Yidan Zhang, Dongxia Pan, Yuelong Huang, Biyun Chen, Li Yin, Donghui Jin, Huilin Liu, Zhongxi Fu, Qiaohua Xu, Xin Xu, Hao Zhang, Youping Xiong, Huajun Long, Xianzhi Li, Libo Zhang, Zhe Qiu

**Affiliations:** a Clinical Trial Service Unit and Epidemiological Studies Unit, Nuffield Department of Population Health, University of Oxford, Oxford, UK; b Medical Research Council Population Health Research Unit, Nuffield Department of Population Health, University of Oxford, Oxford, UK; c Chinese Academy of Medical Sciences, Beijing, China; d Qingdao Municipal Center for Disease Control and Prevention, Qingdao, China; e National Center for Food Safety Risk Assessment, Beijing, China; f Department of Epidemiology and Biostatistics, School of Public Health, Peking University Health Science Center, Beijing, China

## Abstract

**Background:**

The age-specific association between blood pressure and vascular disease has been studied mostly in high-income countries, and before the widespread use of brain imaging for diagnosis of the main stroke types (ischaemic stroke and intracerebral haemorrhage). We aimed to investigate this relationship among adults in China.

**Methods:**

512 891 adults (59% women) aged 30–79 years were recruited into a prospective study from ten areas of China between June 25, 2004, and July 15, 2008. Participants attended assessment centres where they were interviewed about demographic and lifestyle characteristics, and their blood pressure, height, and weight were measured. Incident disease was identified through linkage to local mortality records, chronic disease registries, and claims to the national health insurance system. We used Cox regression analysis to produce adjusted hazard ratios (HRs) relating systolic blood pressure to disease incidence. HRs were corrected for regression dilution to estimate associations with long-term average (usual) systolic blood pressure.

**Findings:**

During a median follow-up of 9 years (IQR 8–10), there were 88 105 incident vascular and non-vascular chronic disease events (about 90% of strokes events were diagnosed using brain imaging). At ages 40–79 years (mean age at event 64 years [SD 9]), usual systolic blood pressure was continuously and positively associated with incident major vascular disease throughout the range 120–180 mm Hg: each 10 mm Hg higher usual systolic blood pressure was associated with an approximately 30% higher risk of ischaemic heart disease (HR 1·31 [95% CI 1·28–1·34]) and ischaemic stroke (1·30 [1·29–1·31]), but the association with intracerebral haemorrhage was about twice as steep (1·68 [1·65–1·71]). HRs for vascular disease were twice as steep at ages 40–49 years than at ages 70–79 years. Usual systolic blood pressure was also positively associated with incident chronic kidney disease (1·40 [1·35–1·44]) and diabetes (1·14 [1·12–1·15]). About half of all vascular deaths in China were attributable to elevated blood pressure (ie, systolic blood pressure >120 mm Hg), accounting for approximately 1 million deaths (<80 years of age) annually.

**Interpretation:**

Among adults in China, systolic blood pressure was continuously related to major vascular disease with no evidence of a threshold down to 120 mm Hg. Unlike previous studies in high-income countries, blood pressure was more strongly associated with intracerebral haemorrhage than with ischaemic stroke. Even small reductions in mean blood pressure at a population level could be expected to have a major impact on vascular morbidity and mortality.

**Funding:**

UK Wellcome Trust, UK Medical Research Council, British Heart Foundation, Cancer Research UK, Kadoorie Charitable Foundation, Chinese Ministry of Science and Technology, and the National Science Foundation of China.

## Introduction

In 2013, WHO set global targets for the control of non-communicable diseases, including a 25% relative reduction in the prevalence of elevated blood pressure by 2025.^1^ About 80% of premature deaths from non-communicable diseases occur in low-income and middle-income countries.^2^ However, estimates of the effects of elevated blood pressure on the worldwide burden of non-communicable diseases, such as ischaemic heart disease and stroke, are based largely on findings of prospective studies in high-income countries.^3^ Studies assessing the hazards of elevated blood pressure in low-income and middle-income countries, including China, are needed to inform local and regional disease prevention strategies. Results of such studies might also substantially improve the global estimates of the burden of disease attributable to elevated blood pressure.

Meta-analyses of prospective studies have demonstrated the importance of moderate differences in blood pressure for risk of ischaemic heart disease and stroke,^4^, ^5^ and randomised trials of blood-pressure-lowering medication have confirmed the reversibility of most of the excess risks within 4–5 years of initiating treatment.^6^ In China, however, despite high rates of vascular mortality (particularly from stroke),^7^ the strengths of the associations between blood pressure and the main types of vascular disease have not been well established; large-scale prospective studies, with measurement of long-term average (ie, usual) levels of blood pressure and reliable phenotyping of the main stroke types (ischaemic stroke and intracerebral haemorrhage), have only become feasible in China over the past decade or so.

**Research in context**

**Evidence before this study**

We did a literature search to identify articles from prospective studies in China that reported on the association between blood pressure and chronic disease. We searched PubMed for articles published between Jan 1, 1960, and March 1, 2017, using the terms “blood pressure” (or “hypertension”) and “prospective study” (or “cohort study” or “longitudinal study”) and “China” (or “Chinese”). We used search terms in English but did not apply any language restrictions. The reference lists of relevant articles were also reviewed. We identified numerous small prospective studies (<50 000 participants) but few large studies, and the largest of these had less than half the number of vascular events than this study. All identified studies reported positive associations between blood pressure and vascular disease (and several reported positive associations with chronic kidney disease and cancer), but most examined effects on mortality only and did not correct for regression dilution bias. Furthermore, the larger prospective studies tended to pre-date the routine use of brain imaging required to reliably differentiate the main stroke types (ischaemic stroke and intracerebral haemorrhage). As such, although blood pressure is an established cause of vascular disease, uncertainty remained about the strength of its association with the major types of vascular disease in China.

**Added value of this study**

This large prospective study reliably quantifies the age-specific associations between blood pressure and major vascular and non-vascular chronic diseases in men and women in China. For each decade of age at risk (40–79 years), there were positive, log-linear associations between systolic blood pressure and risk of ischaemic heart disease, ischaemic stroke, intracerebral haemorrhage, and the aggregate of all major vascular diseases, with no evidence of a threshold down to at least 120 mm Hg. Overall, each 10 mm Hg higher usual systolic blood pressure was associated with an approximately 30% higher risk of ischaemic heart disease and of ischaemic stroke, but the association with intracerebral haemorrhage was about twice as steep. Usual systolic blood pressure was also positively associated with chronic kidney disease and diabetes, but not with cancer (overall or with several common types), liver cirrhosis, or chronic obstructive pulmonary disease; the causal relevance of these positive associations with non-vascular chronic disease remains unclear.

**Implications of all the available evidence**

Our study showed stronger associations between blood pressure and vascular disease than have previous prospective studies in China. The magnitude of the age-specific associations between blood pressure and ischaemic heart disease was similar to those reported in previous large meta-analyses of prospective studies. In contrast to these meta-analyses, however, the present study found strong evidence that, throughout middle age and into old age, there were steeper associations for intracerebral haemorrhage than ischaemic stroke. It was estimated that about half of all vascular deaths in China were attributable to elevated blood pressure (ie, systolic blood pressure >120 mm Hg), accounting for around 1 million deaths (age <80 years) annually; given the strength of the associations between blood pressure and vascular disease, even small reductions in mean blood pressure at a population level would be expected to have a major impact on vascular morbidity and mortality.

Reliable classification of the main stroke types requires brain imaging,^8^ but such imaging is sometimes not performed or is not readily available for epidemiological research. Meta-analyses of prospective studies in western (mainly European and North American) countries, with cohorts that largely pre-date the routine use of brain imaging, report similar associations between systolic blood pressure and risk of both stroke types.^4^ By contrast, several studies done in east Asia (where haemorrhagic stroke is more common than in western populations) have reported more extreme associations with intracerebral haemorrhage than ischaemic stroke.^5^, ^9^, ^10^, ^11^ Few large-scale prospective studies, however, have achieved the high rates of brain imaging required to avoid substantial misclassification between stroke types.

Hence, although blood pressure is an established cause of vascular disease, uncertainty remains about the strength of its association with the main stroke types and with ischaemic heart disease in China. Furthermore, studies in western populations have reported strong positive associations between blood pressure and risk of several non-vascular diseases, including chronic kidney disease, diabetes, and certain cancers, but such associations have not been reliably assessed in China.^12^, ^13^, ^14^ Using data from the large nationwide China Kadoorie Biobank (CKB) prospective study, we aimed to assess the shape and strength of the associations between usual systolic blood pressure and major vascular diseases, including ischaemic heart disease, ischaemic stroke, and intracerebral haemorrhage; to compare these associations with those of usual systolic blood pressure with incident non-vascular chronic diseases; and to estimate the burden of vascular deaths attributable to elevated blood pressure in China.

## Methods

### Study population

Details of the study design and survey methods have been reported previously.^15^, ^16^ Briefly, 512 891 adults (aged 30–79 years) were recruited from the general population in ten areas of China between June 25, 2004, and July 15, 2008. Approximately 30% of the resident population in each area is estimated to have participated in the study. Participants attended temporary assessment centres where interviewers recorded their age, sex, socioeconomic status, aspects of lifestyle (including alcohol intake, smoking, diet, and physical activity), and medical history. Measurements of blood pressure, height, and weight were recorded, and a blood sample was collected. Ethics approval was obtained from the relevant local, national, and international ethics committees, and all participants provided written informed consent.

### Measurement of blood pressure

After participants had sat for at least 5 min, blood pressure was measured twice using a UA-779 digital sphygmomanometer (A&D Instruments; Abingdon, UK). If the difference between the two measurements was more than 10 mm Hg for systolic blood pressure, a third measurement was taken. Only the last two readings were recorded, and the mean of these readings was used in all analyses. Sphygmomanometers were supplied centrally, calibrated daily, and only used by trained fieldworkers. Repeat measurements of blood pressure were obtained from a random sample of 19 788 participants (approximately 5%) about 3 years after baseline (between May 26, and Oct 10, 2008) using procedures identical to those used in the baseline survey. Repeated blood pressure measures were used to correct prospective analyses for the regression dilution bias that results from the inaccuracy with which a single measurement of an exposure at baseline characterises an individual's usual level.^17^ Rosner's regression method was used to calculate the proportional reduction in the strength of the associations that resulted from regression dilution (the regression dilution ratio) for systolic blood pressure, whereby the ratio is equal to the slope of the regression line between baseline and resurvey systolic blood pressure values.^18^

### Morbidity and mortality follow-up

Deaths were identified by electronic linkage to local mortality records, supplemented by annual reviews of local residential registers to check for completeness. Non-fatal events were identified from local chronic disease registries (for stroke, ischaemic heart disease, cancer, and diabetes) and from claims to the national health insurance system, which has almost universal coverage in all study areas. The underlying causes of death and hospital diagnoses were coded in accordance with the International Classification of Diseases 10th revision (ICD-10; see appendix p 3 for ICD-10 codes used to define disease endpoints). Residential records were used to identify participants who moved out of the study areas and were lost to follow-up.

### Statistical analysis

We excluded participants with missing information on blood pressure or any covariates, those with extreme values of systolic blood pressure (<80 mm Hg or ≥250 mm Hg) or diastolic blood pressure (<40 mm Hg or ≥150 mm Hg), those with no follow-up in the age range 40–79 years, and those with a (self-reported) history of vascular disease at baseline (myocardial infarction, stroke, or transient ischaemic attack). For prospective analyses of each non-vascular disease, participants with a history of the relevant disease at baseline were excluded (diabetes at baseline includes both self-reported and screen-detected cases; appendix p 4). The main analyses are of disease associations with systolic blood pressure, but the corresponding analyses for diastolic blood pressure are provided in the appendix pp 17–20.

We used Cox regression analysis to produce adjusted hazard ratios (HRs) for the associations between systolic blood pressure and first occurrence (non-fatal or fatal) of major vascular disease (defined as first myocardial infarction, first stroke, or vascular death), chronic kidney disease, type 2 diabetes, cancer, liver cirrhosis, or chronic obstructive pulmonary disease. Major vascular disease was further classified into ischaemic heart disease (first myocardial infarction or other ischaemic heart disease death), stroke types (ischaemic stroke, intracerebral haemorrhage, and other stroke), and other vascular diseases.

HRs were adjusted for sex, education, area, smoking, alcohol intake, and body-mass index (BMI). An additional term was fitted that allowed the HR in each decade of age to be estimated as the geometric mean of the HRs in the first and second half of that decade, which avoids any assumptions as to whether the proportional associations of risk to usual systolic blood pressure are similar in different decades of age. The log HRs for a 10 mm Hg higher systolic blood pressure were obtained by fitting systolic blood pressure as a continuous variable in men and women separately for each decade of age at risk (40–49, 50–59, 60–69, and 70–79 years). Age-specific and sex-specific HRs were then corrected for age-specific and sex-specific regression dilution ratios to give the HR for a 10 mm Hg higher usual systolic blood pressure (appendix pp 5–7), and combined by calculating their inverse-variance weighted average.

In categorical analyses, participants were divided into five groups according to baseline systolic blood pressure (80–124 mm Hg, 125–144 mm Hg, 145–164 mm Hg, 165–184 mm Hg, and 185–249 mm Hg). HRs were calculated relative to the lowest systolic blood pressure group, and plotted against mean usual systolic blood pressure in each of these groups (appendix p 7); the 95% CIs for these HRs were estimated with the variance of the log risk.^19^ Fatal events were defined as death from any cause within 28 days of the event.

Assuming causality of the association between blood pressure and vascular mortality, we estimated the fraction of deaths attributable to elevated systolic blood pressure by P_d_ × (RR – 1)/RR, where P_d_ represents the proportion of all vascular deaths in a given systolic blood pressure group (with the same systolic blood pressure groups already described) and RR, the relative risk, is approximated by the group-specific HR (this approximation is valid early in follow-up, when the event rate is low) relative to the lowest systolic blood pressure group.^20^ The overall fraction of vascular deaths attributable to elevated systolic blood pressure was calculated as the sum of the group-specific fractions. All analyses were done with SAS software (version 9.3), and figures were plotted using R software (version 3.3).

### Role of the funding source

The funders of the study had no role in study design, data collection, data analysis, data interpretation, or writing of the report. BL, SL, RCl, ZC, and LL had full access to the data and analyses and had final responsibility for the decision to submit for publication.

## Results

Of 512 891 participants, we excluded two with missing information, 110 with extreme systolic blood pressure, 62 with extreme diastolic blood pressure, 488 without follow-up in the age range 40–79 years, and 23 104 with a baseline history of vascular disease. By Jan 1, 2016, 37 289 (7·3%) participants had died and 4875 (1·0%) participants were lost to follow-up.

Among 489 125 participants included in the main analyses, the mean age at baseline was 51 years (SD 10) and 289 224 (59·1%) were women (table). Overall, 211 085 (43·2%) participants resided in urban areas and 240 380 (49·1%) had been educated to middle school or higher. Mean BMI was 23·6 kg/m^2^ (SD 3·4), and 26 393 (5·4%) participants had diabetes (13 300 [2·7%] were clinically diagnosed and a further 13 093 [2·7%] were screen detected from blood glucose tests at baseline). Among men, 123 902 (62·0%) were current smokers and 67 693 (33·9%) consumed alcohol at least weekly, but only 6616 (2·3%) women were smokers and 6017 (2·1%) consumed alcohol at least weekly. Overall, mean systolic blood pressure was 130·6 mm Hg (SD 21·0), and 17 623 (3·6%) participants reported current use of blood-pressure-lowering medication.

**Table:** Baseline characteristics

	**Baseline systolic blood pressure**					**All (n=489 125)**
	80–124 mm Hg (n=158 958)	125–144 mm Hg (n=224 885)	145–164 mm Hg (n=71 081)	165–184 mm Hg (n=24 952)	185–249 mm Hg (n=9249)	
Age (years)	47 (10)	51 (10)	56 (10)	58 (10)	59 (9)	51 (10)
Female	105 630 (66·5%)	123 152 (54·8%)	39 748 (56·7%)	14 905 (59·7%)	5789 (62·6%)	289 224 (59·1%)
Systolic blood pressure (mm Hg)	109·7 (7·3)	130·8 (6·9)	153·3 (5·7)	172·9 (5·6)	197·8 (11·9)	130·6 (21·0)
Diastolic blood pressure (mm Hg)	69·1 (7·0)	78·3 (7·9)	86·4 (9·5)	92·4 (10·7)	100·1 (12·2)	77·7 (11·1)
Body-mass index (kg/m^2^)	22·6 (3·0)	23·8 (3·3)	24·6 (3·5)	24·7 (3·7)	24·8 (3·7)	23·6 (3·4)
MET-h/day of physical activity	22·3 (13·6)	22·0 (14·1)	19·7 (13·9)	18·6 (13·4)	17·8 (12·8)	21·5 (13·9)
Urban area	77 932 (49·0%)	91 615 (40·7%)	29 194 (40·9%)	9276 (37·2%)	3068 (33·2%)	211 085 (43·2%)
Educated to middle school or higher*	93 691 (58·9%)	109 894 (48·9%)	26 815 (37·7%)	7590 (30·4%)	2390 (25·8%)	240 380 (49·1%)
Current smokers (male only†)	35 090 (65·8%)	62 845 (61·8%)	18 053 (57·6%)	5849 (58·2%)	2065 (59·7%)	123 902 (62·0%)
Alcohol consumption at least weekly (male only†)	15 507 (29·1%)	35 539 (34·9%)	11 712 (37·4%)	3637 (36·2%)	1298 (37·5%)	67 693 (33·9%)
Fresh fruit consumed daily	35 367 (22·2%)	38 588 (17·2%)	11 077 (15·6)	3314 (13·3%)	1044 (11·3%)	89 390 (18·3%)
Fresh vegetable consumed daily	150 054 (94·4%)	213 669 (95·0%)	67 361 (94·8%)	23 474 (94·1%)	8609 (93·1%)	463 167 (94·7%)
Diabetes‡	4197 (2·6%)	11 813 (5·3%)	6615 (9·3%)	2695 (10·8%)	1073 (11·6%)	26 393 (5·4%)
Taking blood-pressure-lowering medication	929 (0·6%)	6399 (2·8%)	5890 (8·3%)	3068 (12·3%)	1337 (14·5%)	17 623 (3·6%)

Data are mean (SD) and n (%). The main analysis excludes people with no follow-up at ages 40–79 years; those with a previous diagnosis of vascular disease at baseline (myocardial infarction, stroke, or transient ischaemic attack); those with out-of-range baseline systolic or diastolic blood pressure; and those with missing information on blood pressure or other important factors. MET=metabolic equivalent.

* Middle school is typically attended between ages of 12 years and 15 years.

† 6616 (2·3%) women smoked and 6017 (2·1%) reported drinking alcohol at least weekly.

‡ Includes those with a (self-reported) previous medical diagnosis of diabetes and those detected by blood glucose tests at baseline.

**Figure 1:**
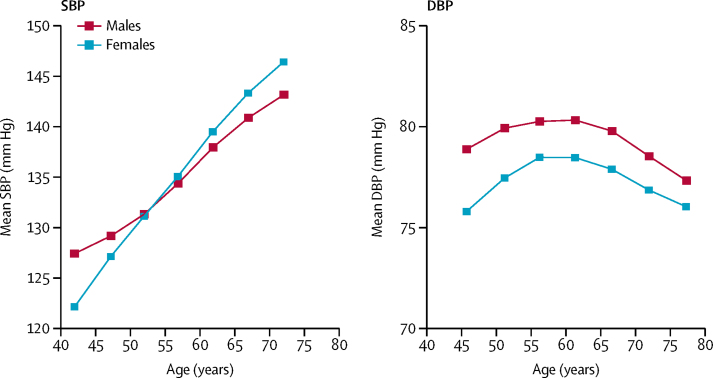
Mean blood pressure at baseline, by age and sex Means standardised for region. Analyses in 489 125 participants; exclusions as in the table. SBP=systolic blood pressure. DBP=diastolic blood pressure.

Mean systolic blood pressure increased linearly with age in both sexes (figure 1). Mean diastolic blood pressure also increased with age until about 50 years, but decreased thereafter. Systolic blood pressure was positively associated with BMI (about 8 mm Hg higher systolic blood pressure per 5 kg/m^2^ higher BMI), and was inversely associated with level of education and residing in an urban area. At resurvey, about 3 years after the baseline survey, the overall regression dilution ratio for systolic blood pressure was 0·6, but it was slightly lower (ie, reflecting greater within-person variability) in men than women, and in older than in younger individuals (appendix p 6).

During 3·9 million person-years of follow-up (median 9 years [IQR 8–10]), 42 662 incident major vascular disease events occurred, including 4450 cases of ischaemic heart disease, 29 343 of ischaemic stroke, 6011 of intracerebral haemorrhage, 1572 of other (or unspecified) stroke, and 1286 of other vascular events. About 90% of stroke cases were diagnosed for stroke type with brain imaging (CT scan or MRI), as estimated from a detailed review of the medical records of a representative subset of cases. Additionally, there were 21 044 incident cases of cancer, 12 241 of diabetes, 8478 of chronic obstructive pulmonary disease, 1940 of chronic kidney disease, and 1740 of chronic liver disease.

**Figure 2:**
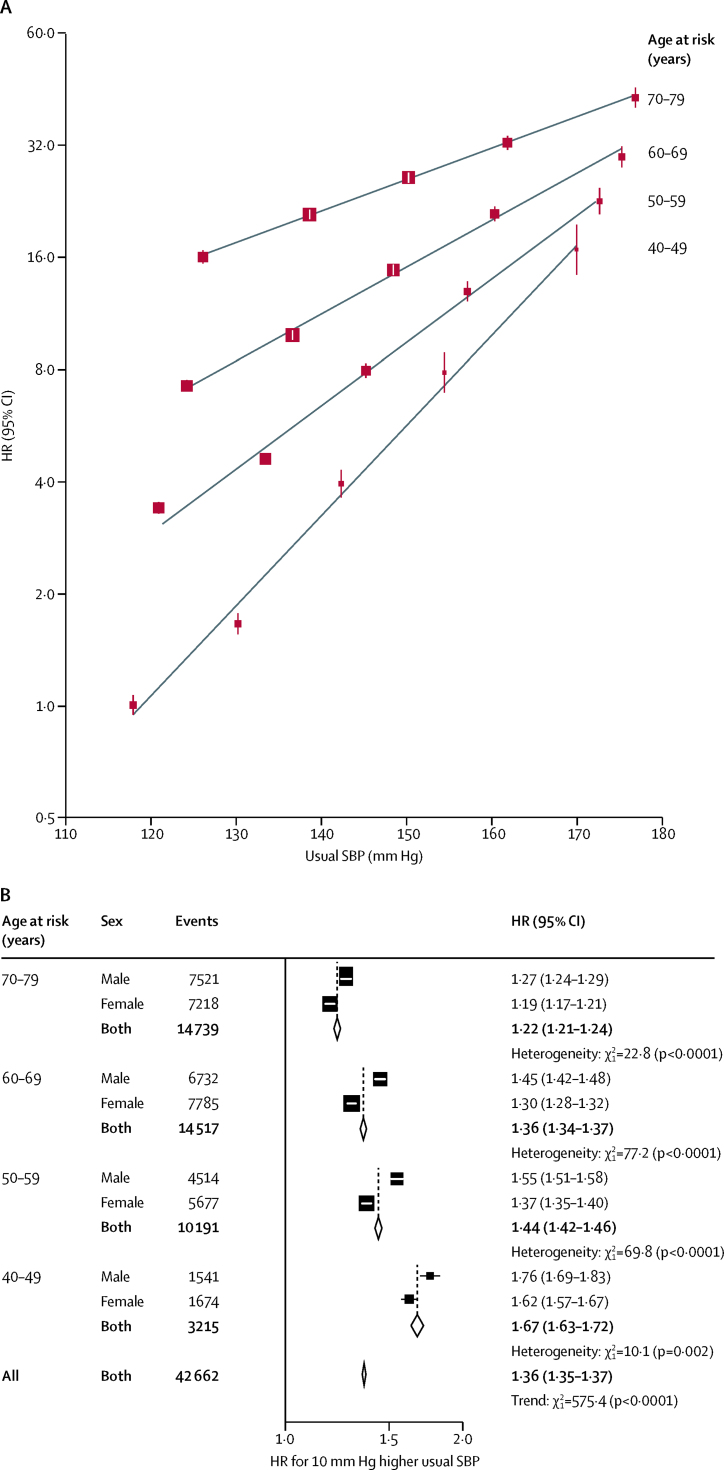
Age-specific incidence of major vascular disease versus usual SBP HRs are adjusted for age at risk (5-year groups), sex, area, education, smoking, alcohol consumption, and body-mass index. (A) The area of each square is inversely proportional to the variance of the category-specific log risk. (B) The area of each square is inversely proportional to the variance of the log HR. Corresponding 95% CIs are plotted as lines. Analyses were done in 489 125 participants at risk and reasons for exclusion are shown in the table. SBP=systolic blood pressure. HR=hazard ratio.

For each decade of age at risk, usual systolic blood pressure was positively and log-linearly associated with risk of major vascular disease throughout the systolic blood pressure range examined, with no evidence of a threshold down to at least 120 mm Hg (figure 2). The HRs were about twice as steep at ages 40–49 years than 70–79 years and, within each decade, were somewhat greater in men than women. Overall (mean age at event 64 years [SD 9]), each 10 mm Hg higher usual systolic blood pressure was associated with 36% higher risk (HR 1·36 [95% CI 1·35–1·37]) of major vascular disease.

**Figure 3:**
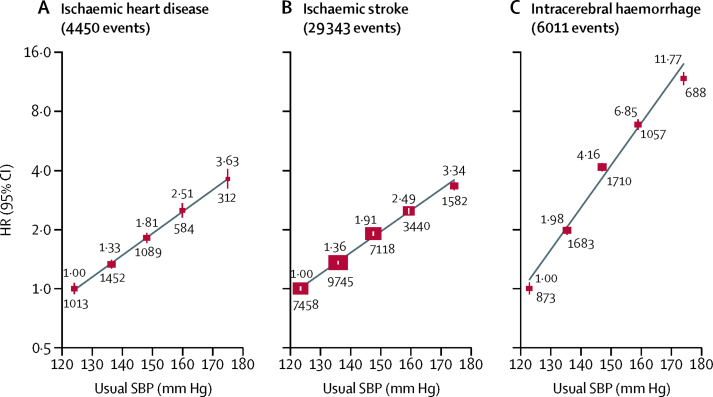
Incidence of ischaemic heart disease, ischaemic stroke, and intracerebral haemorrhage versus usual SBP HRs at ages 40–79 years, adjusted for age at risk (5-year groups), sex, area, education, smoking, alcohol consumption, and body-mass index. For each category, the area of each square is inversely proportional to the variance of the category-specific log risk, which also determines the 95% CI. The HR is shown above each square and numbers of events below. Analyses were done in 489 125 participants at risk and reasons for exclusion are shown in the table. SBP=systolic blood pressure. HR=hazard ratio.

**Figure 4:**
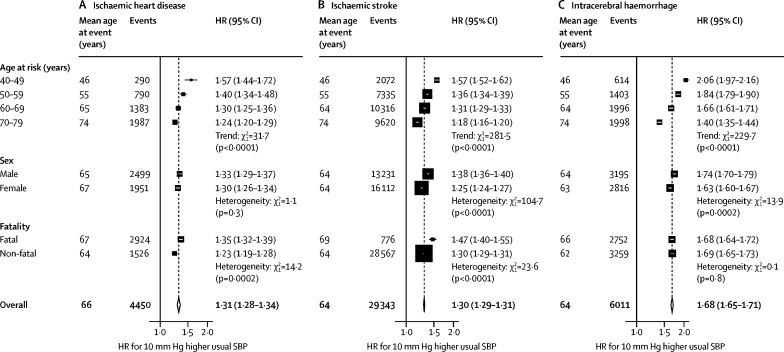
Effect of 10 mm Hg higher SBP on incidence of ischaemic heart disease, ischaemic stroke, and intracerebral haemorrhage, by age and sex HRs for 10 mm Hg higher usual SBP at ages 40–79 years, adjusted for age at risk (5-year groups), sex, area, education, smoking, alcohol consumption, and body-mass index. For each category, area of square is inversely proportional to the variance log HR, which also determines the 95% CI. Analyses were done in 489 125 participants at risk and reasons for exclusion are shown in the table. SBP=systolic blood pressure. HR=hazard ratio.

The associations between usual systolic blood pressure and ischaemic heart disease, ischaemic stroke, and intracerebral haemorrhage were also approximately log-linear throughout the systolic blood pressure range examined (figure 3). Overall, each 10 mm Hg higher usual systolic blood pressure was associated with about 30% higher risk of ischaemic heart disease (HR 1·31 [95% CI 1·28–1·34]) and ischaemic stroke (1·30 [1·29–1·31]), but the associations with intracerebral haemorrhage were about twice as steep (1·68 [1·65–1·71]). The HRs for each of these vascular diseases were substantially greater at ages 40–49 years than 70–79 years (figure 4; appendix p 8). The associations were also somewhat greater in men than women for both stroke types, but not for ischaemic heart disease. In addition, associations were greater for fatal than non-fatal ischaemic heart disease (1·35 [1·32–1·39] *vs* 1·23 [1·19–1·28]) and for fatal than non-fatal ischaemic stroke (1·47 [1·40–1·55] *vs* 1·30 [1·29–1·31]), but there were no such differences for intracerebral haemorrhage.

**Figure 5:**
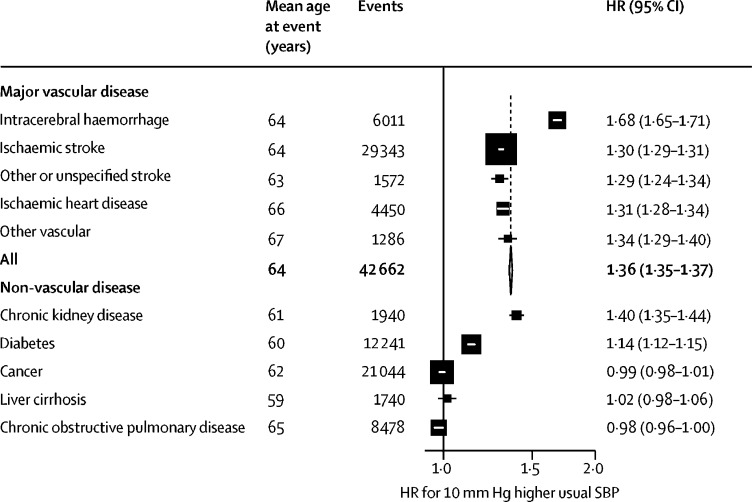
Effect of 10 mm Hg higher SBP on incidence of vascular and non-vascular chronic disease HRs for 10 mm Hg higher usual SBP at ages 40–79 years, adjusted for age at risk (5-year groups), sex, area, education, smoking, alcohol consumption, and body-mass index. For each category, the area of the square is inversely proportional to the variance of the log HR, which also determines the 95% CI. Reasons for exclusion are shown in the table; analyses of each non-vascular disease further exclude participants with a previous diagnosis of the relevant disease at baseline (appendix p 4). SBP=systolic blood pressure. HR=hazard ratio.

Only 4% of strokes were classified as other or unspecified type, and the strength of these associations with usual systolic blood pressure was similar to that for ischaemic stroke (figure 5; appendix p 9). For the aggregate of all other vascular events, the strength of the association was consistent with all major vascular disease. Usual systolic blood pressure was also strongly and positively associated with risk of chronic kidney disease (HR 1·40 [95% CI 1·35–1·44]) and less strongly associated with diabetes (1·14 [1·12–1·15]), but was not associated with cancer, liver cirrhosis, or chronic obstructive pulmonary disease.

For vascular mortality at ages 40–79 years, each 10 mm Hg higher usual systolic blood pressure was associated with about a 50% higher risk of vascular death (HR 1·49 [95% CI 1·47–1·51]; mean age at death 68 years [SD 9]; appendix p 10). Non-vascular mortality was also positively associated with usual systolic blood pressure, although the risks were much weaker than those for vascular mortality 1·07 [1·06–1·08]). Assuming causality, the excess vascular mortality associated with elevated blood pressure (ie, systolic blood pressure >120 mm Hg) accounted for about half (45% [41–48%]) of all vascular deaths (ie, roughly 1·1 million deaths) between ages 40 years and 79 years in China in 2015 (appendix p 11).

In sensitivity analyses (appendix pp 12–16), the associations were similar in urban and rural areas (except those for ischaemic stroke, which were somewhat shallower in urban than rural areas), and were not materially altered by further adjustment for other potential confounders (including physical activity, frequency of fruit and vegetable consumption, and, where appropriate, diabetes); excluding the first 3 years of follow-up (to assess for reverse causality); excluding participants who were using blood-pressure-lowering medication at baseline; or censoring at the first occurrence of chronic disease of any type (to assess for competing risks). Furthermore, there was no evidence that the associations varied by type of chronic kidney disease or by type of common cancer. The associations for diastolic blood pressure were broadly consistent with those for systolic blood pressure: there were log-linear associations of usual diastolic blood pressure with each of the major types of vascular disease throughout the blood pressure range examined (75–100 mm Hg usual diastolic blood pressure), and the strengths of these associations for 5 mm Hg higher usual diastolic blood pressure were equivalent to about 10 mm Hg higher usual systolic blood pressure (appendix pp 17–20).

## Discussion

In this Chinese population, usual blood pressure was positively and log-linearly associated with risk of ischaemic heart disease, ischaemic stroke, intracerebral haemorrhage, and the aggregate of all major vascular diseases. These relationships continued down to at least 120 mm Hg systolic blood pressure and 75 mm Hg diastolic blood pressure, below which the evidence was scarce. The associations attenuated with increasing age, but even at ages 70–79 years there were strong associations with each of the main types of vascular disease. At all ages, there were steeper associations between blood pressure and intracerebral haemorrhage than ischaemic stroke. We estimated that about half of all premature vascular deaths were attributable to elevated blood pressure (ie, systolic blood pressure >120 mm Hg).

Our study showed stronger associations between blood pressure and vascular disease than have previous prospective studies in China, largely because those studies did not correct for within-person variability in blood pressure (regression dilution bias).^21^, ^22^, ^23^ Compared with previous large meta-analyses of prospective studies that corrected for regression dilution (the Prospective Studies Collaboration^4^ and the Asia Pacific Cohort Studies Collaboration^5^), the findings are consistent for ischaemic heart disease but differ somewhat for stroke types. By contrast with these meta-analyses, our study found strong evidence that, throughout middle age and into old age, there were steeper associations for intracerebral haemorrhage than ischaemic stroke.

These differences might, in part, reflect the high rate of brain imaging in this study. The use of brain imaging for diagnosis of stroke types was less common before the mid-1990s and, hence, it is possible that there was substantial misclassification of stroke types in some of the older studies included in these meta-analyses. By contrast, brain imaging was used to diagnose stroke types in about 90% of stroke cases in the CKB study. The greater use of brain imaging might also have allowed more non-fatal strokes to be identified than previously. There were shallower associations for non-fatal than fatal ischaemic stroke (unlike for intracerebral haemorrhage), and there is some evidence that both case fatality and the strength of associations with blood pressure might differ between the different aetiological subtypes of ischaemic stroke (large-artery atherosclerosis, small-vessel disease, cardioembolism, or other pathology).^8^, ^24^ Information on subtypes of ischaemic stroke is not yet available in the present study, but small-vessel (lacunar) infarcts, with low case fatality, might well be more common in Chinese than white populations.^25^

Randomised controlled trials^6^, ^26^ of blood-pressure-lowering medication have shown the reversibility of some of the excess vascular risks associated with elevated blood pressure. A comparison of the findings from blood-pressure-lowering trials with the predicted effects of blood pressure from prospective cohort studies suggests that about 60% of the predicted risks of ischaemic heart disease and 80% of the predicted risks of stroke were reversed within 4–5 years of initiating treatment.^26^ Furthermore, the trial results indicate that the proportional effects of blood pressure on vascular risk were similar among people with and without cardiovascular disease, so the absolute benefits are very much greater among those with existing disease.^6^ Similarly, the absolute benefits of blood pressure lowering are likely to be greater in older than younger adults, given the greater absolute risk of vascular disease at older age.

Guidelines on the management of hypertension generally recommend initiating treatment in adults with an average systolic blood pressure of 140 mm Hg or more, or diastolic blood pressure of 90 mm Hg or more, and some recent randomised trials, in selected populations, have found benefit in initiating treatment at lower levels.^27^, ^28^ However, the use of blood-pressure-lowering treatment in China is much lower than in western populations.^29^ Our previous analyses of the CKB study indicate that, consistent with nationally representative surveys in China,^30^, ^31^ about a third of adults had hypertension at baseline (defined as systolic blood pressure ≥140 mm Hg, or diastolic blood pressure ≥90 mm Hg, or receiving treatment for hypertension).^32^ Of those with hypertension, about a third were diagnosed; of those diagnosed, about half were treated; and, of those treated, about a third had their hypertension controlled. The result was that less than 5% of participants with hypertension achieved properly controlled blood pressure.

Previous studies reported associations between blood pressure variability and risk of vascular disease independently of baseline systolic blood pressure,^33^, ^34^ but there were an insufficient number of repeat blood pressure measures to assess any such effects in this study. However, other studies have suggested that, after correction for regression dilution bias in blood pressure variability, the effects of any such variability in systolic blood pressure on risk of vascular diseases, independent of usual levels of systolic blood pressure, are likely to be modest.^35^

We observed significant positive associations between systolic blood pressure and chronic kidney disease and diabetes, but the causal relevance of these associations remains uncertain. Neither association is strongly supported by evidence from trials, and might well be accounted for by reverse causality or residual confounding. A large meta-analysis^6^ of randomised controlled trials of blood-pressure-lowering medications reported that lowering blood pressure had no significant effect on the incidence of renal failure (10 mm Hg lower systolic blood pressure was associated with RR 0·95 [95% CI 0·84–1·07]). However, renal failure might not be entirely consistent with chronic kidney disease as defined in this study (the level of renal impairment among incident chronic kidney disease cases was not available). For diabetes, there is some evidence from randomised trials that lower blood pressure is associated with a reduced incidence of diabetes, but the effects are limited to certain medications only (angiotensin-converting-enzyme inhibitors and angiotensin receptor blockers), indicating that the renin–angiotensin system might be causally related to diabetes rather than actual levels of blood pressure.^36^

The chief strengths of this study include the large number of disease events, high-quality measurements of blood pressure (including the training of technicians), and long-term follow-up of a wide range of clinically validated disease outcomes. The main findings support efforts to address the substantial burden of blood pressure on vascular disease in China, and indicate that even small reductions in mean blood pressure at a population level can have a substantial effect on disease risk: for example, 5 mm Hg lower usual blood pressure would prevent around 350 000 deaths per year among individuals younger than 80 years of age (and around 200 000 deaths at <70 years). The use of blood-pressure-lowering medication is low in China, and strategies to improve rates of awareness, detection, and treatment of individuals with hypertension are likely to be highly cost-effective, especially among those with existing vascular disease.^37^ In the absence of well developed primary care systems, population-based approaches are also required to address the major determinants of elevated blood pressure in China, including high sodium intake (eg, through salt reduction), high alcohol consumption, excess adiposity, lack of regular physical activity, and poor home heating in winter.^38^, ^39^, ^40^

In conclusion, among men and women in China, higher levels of blood pressure were continuously and positively associated with higher risks of major vascular disease, with no evidence of a threshold down to at least 120 mm Hg systolic blood pressure and 75 mm Hg diastolic blood pressure. Unlike studies in western populations, blood pressure was more strongly associated with intracerebral haemorrhage than ischaemic stroke. There was also evidence of positive associations with some non-vascular chronic diseases, but the causality of these associations remains unclear. It was estimated that about half of all vascular deaths in China were attributable to elevated blood pressure, accounting for roughly 1 million deaths (age <80 years) annually; given the strength of the associations between blood pressure and vascular disease, even small reductions in mean blood pressure at a population level would be expected to have a major impact on vascular morbidity and mortality.

## References

[1] WHO (2013). Global action plan for the prevention and control of noncommunicable diseases 2013–2020.

[2] Benziger CP, Roth GA, Moran AE (2016). The global burden of disease study and the preventable burden of NCD. Glob Heart.

[3] Forouzanfar MH, Liu P, Roth GA (2017). Global burden of hypertension and systolic blood pressure of at least 110 to 115 mm Hg, 1990–2015. JAMA.

[4] Prospective Studies Collaboration (2002). Age-specific relevance of usual blood pressure to vascular mortality: a meta-analysis of individual data for one million adults in 61 prospective studies. Lancet.

[5] Asia Pacific Cohort Studies Collaboration (2003). Blood pressure and cardiovascular disease in the Asia Pacific region. J Hypertens.

[6] Ettehad D, Emdin CA, Kiran A (2016). Blood pressure lowering for prevention of cardiovascular disease and death: a systematic review and meta-analysis. Lancet.

[7] Global Burden of Disease 2016 Causes of Death Collaborators (2017). Global, regional, and national age-sex specific mortality for 264 causes of death, 1980–2016: a systematic analysis for the Global Burden of Disease Study 2016. Lancet.

[8] Hankey GJ (2017). Stroke. Lancet.

[9] Song YM, Sung J, Lawlor DA, Davey Smith G, Shin Y, Ebrahim S (2004). Blood pressure, haemorrhagic stroke, and ischaemic stroke: the Korean national prospective occupational cohort study. BMJ.

[10] Kim HC, Nam CM, Jee SH, Suh I (2005). Comparison of blood pressure-associated risk of intracerebral hemorrhage and subarachnoid hemorrhage: Korea Medical Insurance Corporation study. Hypertension.

[11] Tsai CF, Jeng JS, Anderson N, Sudlow CLM (2017). Comparisons of risk factors for intracerebral hemorrhage versus ischemic stroke in Chinese patients. Neuroepidemiology.

[12] Emdin CA, Anderson SG, Woodward M, Rahimi K (2015). Usual blood pressure and risk of new-onset diabetes: evidence from 4·1 million adults and a meta-analysis of prospective studies. J Am Coll Cardiol.

[13] Stocks T, Van Hemelrijck M, Manjer J (2012). Blood pressure and risk of cancer incidence and mortality in the Metabolic Syndrome and Cancer Project. Hypertension.

[14] Herrington W, Staplin N, Judge PK (2017). Evidence for reverse causality in the association between blood pressure and cardiovascular risk in patients with chronic kidney disease. Hypertension.

[15] Chen Z, Lee L, Chen J (2005). Cohort profile: the Kadoorie Study of Chronic Disease in China (KSCDC). Int J Epidemiol.

[16] Chen Z, Chen J, Collins R (2011). China Kadoorie Biobank of 0·5 million people: survey methods, baseline characteristics and long-term follow-up. Int J Epidemiol.

[17] Clarke R, Shipley M, Lewington S (1999). Underestimation of risk associations due to regression dilution in long-term follow-up of prospective studies. Am J Epidemiol.

[18] Rosner B, Willett WC, Spiegelman D (1989). Correction of logistic regression relative risk estimates and confidence intervals for systematic within-person measurement error. Stat Med.

[19] Plummer M (2004). Improved estimates of floating absolute risk. Stat Med.

[20] Rockhill B, Newman B, Weinberg C (1998). Use and misuse of population attributable fractions. Am J Public Health.

[21] Gu D, Kelly TN, Wu X (2008). Blood pressure and risk of cardiovascular disease in Chinese men and women. Am J Hypertens.

[22] He J, Gu D, Chen J (2009). Premature deaths attributable to blood pressure in China: a prospective cohort study. Lancet.

[23] Zhou M, Offer A, Yang G (2008). Body mass index, blood pressure, and mortality from stroke: a nationally representative prospective study of 212,000 Chinese men. Stroke.

[24] Jackson C, Sudlow C (2005). Are lacunar strokes really different? A systematic review of differences in risk factor profiles between lacunar and nonlacunar infarcts. Stroke.

[25] Tsai CF, Thomas B, Sudlow CL (2013). Epidemiology of stroke and its subtypes in Chinese vs white populations: a systematic review. Neurology.

[26] Law MR, Morris JK, Wald NJ (2009). Use of blood pressure lowering drugs in the prevention of cardiovascular disease: meta-analysis of 147 randomised trials in the context of expectations from prospective epidemiological studies. BMJ.

[27] Whelton PK, Carey RM, Aronow WS (2017). 2017 ACC/AHA/AAPA/ABC/ACPM/AGS/APhA/ASH/ASPC/NMA/PCNA guideline for the prevention, detection, evaluation, and management of high blood pressure in adults: a report of the American College of Cardiology/American Heart Association Task Force on Clinical Practice Guidelines. J Am Coll Cardiol.

[28] Wright JT, Williamson JD, Whelton PK (2015). A randomized trial of intensive versus standard blood-pressure control. N Engl J Med.

[29] Lu J, Lu Y, Wang X (2017). Prevalence, awareness, treatment, and control of hypertension in China: data from 1·7 million adults in a population-based screening study (China PEACE Million Persons Project). Lancet.

[30] Gao Y, Chen G, Tian H (2013). Prevalence of hypertension in china: a cross-sectional study. PLoS One.

[31] Wu Y, Huxley R, Li L (2008). Prevalence, awareness, treatment, and control of hypertension in China: data from the China National Nutrition and Health Survey 2002. Circulation.

[32] Lewington S, Lacey B, Clarke R (2016). The burden of hypertension and associated risk for cardiovascular mortality in China. JAMA Intern Med.

[33] Rothwell PM, Howard SC, Dolan E (2010). Prognostic significance of visit-to-visit variability, maximum systolic blood pressure, and episodic hypertension. Lancet.

[34] Shimbo D, Newman JD, Aragaki AK (2012). Association between annual visit-to-visit blood pressure variability and stroke in postmenopausal women: data from the Women's Health Initiative. Hypertension.

[35] Chang TI, Reboussin DM, Chertow GM (2017). Visit-to-visit office blood pressure variability and cardiovascular outcomes in SPRINT (systolic blood pressure intervention trial). Hypertension.

[36] Elliott WJ, Meyer PM (2007). Incident diabetes in clinical trials of antihypertensive drugs: a network meta-analysis. Lancet.

[37] Gu D, He J, Coxson PG (2015). The cost-effectiveness of low-cost essential antihypertensive medicines for hypertension control in China: a modelling study. PLoS Med.

[38] Brown IJ, Tzoulaki I, Candeias V, Elliott P (2009). Salt intakes around the world: implications for public health. Int J Epidemiol.

[39] Lewington S, Li L, Sherliker P (2012). Seasonal variation in blood pressure and its relationship with outdoor temperature in 10 diverse regions of China: the China Kadoorie Biobank. J Hypertens.

[40] Poulter NR, Prabhakaran D, Caulfield M (2015). Hypertension. Lancet.

