# Supplementary material for: Age-specific association between blood pressure and vascular and non-vascular chronic diseases in 0·5 million adults in China: a prospective cohort study
Source: Lancet Glob Health. 2018 May 14;6(6):e641–9. doi: 10.1016/S2214-109X(18)30217-1 (PMC5960069; doi:10.1016/S2214-109X(18)30217-1)
Supplement: Supplementary appendix [file mmc1.pdf]

THE LANCET
Global Health

Supplementary appendix

This appendix formed part of the original submission and has been peer reviewed. We post it as supplied by the authors.

Supplement to: Lacey B, Lewington S, Clarke R, et al. Age-specific association between blood pressure and vascular and non-vascular chronic diseases in 0·5 million adults in China: a prospective cohort study. *Lancet Glob Health* 2018; **6:** e641–49.

This page intentionally left blank

Age-specific relation of blood pressure to vascular and non-vascular chronic diseases in China: a prospective study of 0.5 million adults

Supplementary Appendix, Table of Contents

	Page
**Table S1:** Chronic disease endpoints and ICD-10 codes	3
**Table S2:** Number of incident vascular and non-vascular chronic disease events included in main analyses, by age at risk and sex	4
**Table S3:** Number of participants and their mean age and blood pressure at baseline, by baseline age and sex	5
**Table S4:** Regression dilution ratios for blood pressure, by age- and sex-specific baseline groups (among 15 853 participants resurveyed)	6
**Table S5:** Estimated usual blood pressure, by age at baseline and sex	7
**Table S6**: Incidence of ischaemic heart disease, ischaemic stroke and intracerebral haemorrhage versus usual SBP, by age at risk and sex	8
**Figure S1:** Incidence of vascular and non-vascular chronic disease versus usual SBP	9
**Figure S2:** Mortality from vascular and non-vascular chronic disease: hazard ratio for 10 mmHg higher usual SBP	10
**Figure S3:** Vascular deaths attributable to elevated SBP in China, 2015	11
**Table S7:** Incidence of vascular and non-vascular chronic disease: hazard ratios for 10 mmHg higher usual SBP, progressively adjusted for potential confounders	12
**Table S8:** Incidence of vascular and non-vascular chronic disease: hazard ratios for 10 mmHg higher usual SBP in urban and rural areas	13
**Table S9:** Incidence of vascular and non-vascular chronic disease: hazard ratios for 10 mmHg higher usual SBP, excluding the first 3 years of follow-up and excluding those taking blood pressure-lowering medication at baseline	14
**Table S10:** Incidence of chronic kidney disease and diabetes versus usual SBP, by age at risk and sex	15
**Table S11:** Incidence of chronic kidney disease and cancer versus usual SBP, by disease subtype	16
**Figure S4:** Incidence of ischaemic heart disease, ischaemic stroke and intracerebral haemorrhage versus usual DBP	17
**Table S12:** Incidence of ischaemic heart disease, ischaemic stroke and intracerebral haemorrhage versus usual DBP, by age at risk and sex	18
**Figure S5:** Incidence of vascular and non-vascular chronic disease: hazard ratio for 5 mmHg higher usual DBP	19
**Figure S6:** Incidence of vascular and non-vascular chronic disease versus usual DBP	20
**China Kadoorie Biobank collaborative group**	21

Table S1: Chronic disease endpoints and ICD-10 codes

Endpoint name	ICD-10 codes*
**Major vascular disease**†	
Ischaemic heart disease‡	
Myocardial infarction	I21, I22, I23
Other ischaemic heart disease death	I20, I24, I25
Stroke	
Ischaemic stroke	I63
Intracerebral haemorrhage	I61
Subarachnoid haemorrhage	I60
Unspecified stroke	I64
Other stroke event§	-
Other vascular	
Other vascular death	I00-I15,I26-I52,I62,I65-I99,R96
Other vascular event‖	-
**Non-vascular disease**	
Chronic kidney disease	E10.2, E11.2, E12.2, E13.2, E14.2, N03, N18, N26, N27, I12, I13
Diabetes (type 2)	E11, E13, E14
Cancer	C00-C97
Liver cirrhosis	K70, K73, K74
COPD	J42-J44

COPD=Chronic obstructive pulmonary disease.
* Source of mortality and morbidity follow-up include: death certificate (underlying cause of death only) and non-fatal events from local chronic disease registries (for ischaemic heart disease, stroke, cancer and diabetes) and from national health insurance claims system.
† Major vascular disease defined as first myocardial infarction, first stroke or other vascular death.
‡ Ischaemic heart disease defined as first myocardial infarction or other ischaemic heart disease death.
§ Type of first stroke (ischaemic stoke, intracerebral haemorrhage or subarachnoid haemorrhage) could not be established.
‖ Type of first major vascular disease event (ischaemic heart disease or stroke) could not be established.

Table S2: Number of incident vascular and non-vascular chronic disease events included in main analyses,* by age at risk and sex

Age at risk, years	Major vascular disease, n[†]						Non-vascular disease, n				
	Ischaemic heart disease[‡]	Ischaemic stroke	Intracerebral haemorrhage	Other or unspecified stroke	Other vascular[§]	All	Cancer	Diabetes (type 2)	COPD	Chronic kidney disease	Liver cirrhosis
**Male**											
40-49	192	941	297	77	34	1541	846	824	258	112	193
50-59	487	3058	667	172	130	4514	2513	1436	704	183	351
60-69	774	4475	1107	207	169	6732	3759	1516	1441	258	287
70-79	1046	4757	1124	220	374	7521	3382	837	1669	264	165
All	2499	13231	3195	676	707	20308	10500	4613	4072	817	996
**Female**											
40-49	98	1131	317	94	34	1674	1838	1069	451	194	122
50-59	303	4277	736	274	87	5677	3200	2686	991	350	264
60-69	609	5841	889	297	149	7785	3274	2602	1498	338	222
70-79	941	4863	874	231	309	7218	2232	1271	1466	241	136
All	1951	16112	2816	896	579	22354	10544	7628	4406	1123	744
**Overall**	4450	29343	6011	1572	1286	42662	21044	12241	8478	1940	1740

COPD=Chronic obstructive pulmonary disease.

* Exclusions as in Table 1. For prospective analyses of each non-vascular disease, we further excluded participants with a prior diagnosis of the relevant disease at baseline (ie, for analyses of cancer, we further excluded participants with a diagnosis of cancer at baseline). Following these exclusions, there were 489,125 participants in the analyes for major vascular disease, 486,746 for cancer, 462,732 for diabetes, 454,411 for COPD, 482,322 for chronic kidney disease, and 483,269 for liver cirrhosis.

† First major vascular disease event only. Major vascular disease defined as first myocardial infarction, first stroke or other vascular death.

‡ Ischaemic heart disease defined as first myocardial infarction or other coronary death.

§ Other vascular endpoint includes: 187 hypertensive heart disease deaths, 111 rheumatic heart disease deaths, 108 pulmonary heart disease deaths, 132 cardiac arrest deaths, 103 sudden deaths of unknown cause, 48 heart failure deaths and 597 events of other vascular diseases.

Table S3: Number of participants and their mean age and blood pressure at baseline, by baseline age and sex*

Age at baseline, years	Participants	Mean age, years	Mean (SD) blood pressure at baseline	
			SBP, mmHg	DBP, mmHg
**Male**				
40-49	58413	44	128.5 (17.2)	79.6 (11.4)
50-59	60935	54	132.7 (19.9)	80.2 (11.3)
60-69	37192	64	139.1 (22.1)	79.0 (11.3)
70-79	14091	72	142.8 (22.3)	77.1 (11.2)
All (40-79)	170631	55	133.5 (20.3)	79.5 (11.3)
**Female**				
40-49	92292	44	124.3 (18.7)	76.5 (10.7)
50-59	89641	54	132.7 (21.8)	78.4 (10.9)
60-69	45058	64	140.6 (23.1)	77.3 (10.9)
70-79	14462	72	145.3 (23.7)	75.6 (11.2)
All (40-79)	241453	53	131.7 (22.2)	77.3 (10.9)
**Overall**	412084	54	132.5 (21.4)	78.2 (11.1)

*Exclusions as in Table 1, with further exclusion of those outside the age range 40-79 years at baseline.

Table S4: Regression dilution ratios for blood pressure, by age- and sex-specific baseline groups (among 15 853 participants resurveyed*)

Age at baseline, years	Number of participants	Mean interval between baseline and resurvey measures, years	Regression dilution ratios†	
			SBP	DBP
**Male**				
40-49	2126	2.5	0.61 (0.58-0.65)	0.62 (0.59-0.65)
50-59	2208	2.6	0.59 (0.56-0.62)	0.56 (0.53-0.59)
60-69	1456	2.6	0.54 (0.51-0.57)	0.57 (0.54-0.61)
70-79	470	2.6	0.52 (0.46-0.58)	0.56 (0.49-0.62)
**Female**				
40-49	3536	2.6	0.65 (0.62-0.67)	0.63 (0.61-0.66)
50-59	3711	2.6	0.63 (0.61-0.65)	0.60 (0.58-0.62)
60-69	1850	2.7	0.59 (0.56-0.62)	0.59 (0.56-0.63)
70-79	496	2.6	0.57 (0.52-0.62)	0.57 (0.51-0.63)

* Exclusions as in Table 1, with further exclusion of those outside the age range of 40-79 years at baseline, and those with missing or out of range SBP (<80 or ≥250 mmHg) or DBP (<40 or ≥150 mmHg) at resurvey.

† Calculated using Rosner's regression method.

Table S5: Estimated usual blood pressure,* by age at baseline and sex

Baseline blood pressure group	Usual blood pressure (mmHg), by sex and baseline age							
	Male				Female			
	40-49	50-59	60-69	70-79	40-49	50-59	60-69	70-79
**SBP, mmHg**								
80-125	120.0	121.4	124.0	125.5	116.7	120.6	124.3	126.5
125-144	131.3	133.4	136.2	138.0	129.5	133.4	136.9	139.0
145-164	143.2	145.2	147.9	149.5	141.7	145.3	148.7	150.7
165-184	155.3	157.0	159.8	161.2	153.8	157.2	160.5	162.3
185-249	170.2	172.2	174.9	176.0	169.7	172.8	175.3	177.3
Overall	128.5	132.7	139.1	142.8	124.3	132.7	140.6	145.3
**DBP, mmHg**								
40-75	72.6	73.6	72.7	71.6	70.9	72.2	71.5	70.5
75-84	79.7	79.9	79.3	78.4	78.3	79.1	78.7	77.8
85-94	85.4	85.1	84.7	83.7	84.3	84.7	84.1	83.2
95-104	91.6	90.6	90.2	89.3	90.5	90.5	89.9	88.8
105-149	99.5	97.6	97.0	95.5	98.2	97.7	96.7	96.0
Overall	79.6	80.2	79.0	77.1	76.5	78.4	77.3	75.6

*Usual blood pressure in each baseline blood pressure group was estimated by (A-B) x RDR + A, where A represents the overall age-specific mean blood pressure (Table S3), B is the given baseline blood pressure group mean and RDR is the age- and sex-specific regression dilution ratio (Table S4). For plots of incident chronic disease versus usual blood pressure at age 40-79 years, we calculated the weighted average of the age- and sex-specific usual blood pressure values, with weights equal to the inverse-variance of the age- and sex-specific hazard ratios.

Table S6: Incidence of ischaemic heart disease, ischaemic stroke and intracerebral haemorrhage versus usual SBP, by age at risk and sex

Age at risk, years	Ischaemic heart disease			Ischaemic stroke			Intracerebral haemorrhage		
	Mean age at event	No. of events	Hazard ratio (95% CI)	Mean age at event	No. of events	Hazard ratio (95% CI)	Mean age at event	No. of events	Hazard ratio (95% CI)
**Male**									
40-49	46	192	1.58 (1.40-1.78)	46	941	1.67 (1.58-1.76)	45	297	2.18 (2.02-2.36)
50-59	55	487	1.45 (1.35-1.55)	55	3058	1.48 (1.44-1.52)	55	667	1.93 (1.84-2.03)
60-69	65	774	1.32 (1.24-1.39)	65	4475	1.40 (1.37-1.43)	65	1107	1.76 (1.69-1.84)
70-79	74	1046	1.23 (1.17-1.30)	74	4757	1.22 (1.19-1.25)	74	1124	1.45 (1.39-1.52)
All	65	2499	1.33 (1.29-1.37)	64	13231	1.38 (1.36-1.40)	64	3195	1.74 (1.70-1.79)
**Female**									
40-49	46	98	1.56 (1.37-1.79)	46	1131	1.51 (1.45-1.57)	46	317	1.99 (1.88-2.11)
50-59	55	303	1.36 (1.26-1.46)	55	4277	1.30 (1.27-1.33)	55	736	1.79 (1.72-1.86)
60-69	65	609	1.29 (1.22-1.37)	64	5841	1.26 (1.23-1.28)	64	889	1.56 (1.49-1.62)
70-79	75	941	1.25 (1.19-1.31)	74	4863	1.15 (1.12-1.17)	74	874	1.34 (1.28-1.41)
All	67	1951	1.30 (1.26-1.34)	64	16112	1.25 (1.24-1.27)	63	2816	1.63 (1.60-1.67)
**Overall**	66	4450	1.31 (1.28-1.34)	64	29343	1.30 (1.29-1.31)	64	6011	1.68 (1.65-1.71)

Hazard ratios are adjusted for age at risk (5-year groups), sex (where appropriate), region, education, smoking, alcohol and BMI. Analyses in 489,125 participants at risk; exclusions as in Table 1.

Figure S1: Incidence of vascular and non−vascular chronic disease versus usual SBP

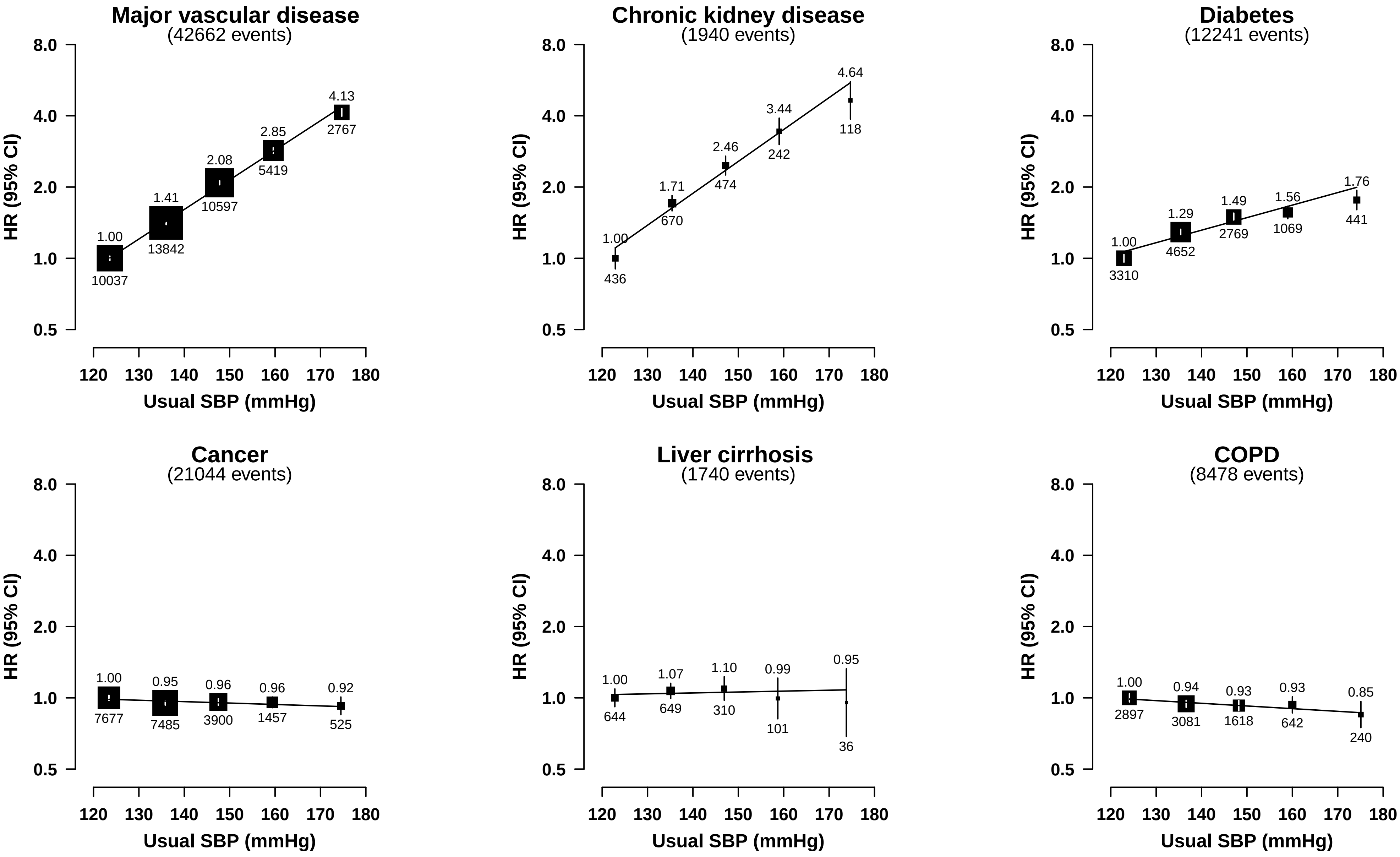

COPD=Chronic obstructive pulmonary disease. Hazard ratios (HR) at age 40−79 years, adjusted for age at risk (5−year groups), sex, region, education, smoking, alcohol and BMI. For each category, the area of each square is inversely proportional to the variance of the category−specific log risk, which also determines the confidence interval (CI). HR is shown above each square and numbers of events below. Exclusions as in Table 1; analyses of each non-vascular disease, further exclude participants with a prior diagnosis of the relevant disease at baseline (appendix p4).

Figure S2: Mortality from Vascular and non−vascular chronic disease: hazard ratio for 10 mmHg higher usual SBP

	Mean age at death	Number of deaths	HR (95% CI)
**ascular mortality**			
Intracerebral haemorrhage	66	3586	1.67 (1.64 − 1.71)
Ischaemic stroke	69	1048	1.49 (1.43 − 1.56)
Ischaemic heart disease	68	3566	1.36 (1.32 − 1.39)
Other or unspecified stroke	68	298	1.44 (1.33 − 1.56)
Other vascular	69	1982	1.41 (1.36 − 1.45)
**All**	**68**	**10480**	**1.49 (1.47 − 1.51)**
**Non−vascular mortality**			
Chronic kidney disease	66	406	1.52 (1.42 − 1.62)
Diabetes	69	116	1.24 (1.08 − 1.43)
Cancer	65	10281	1.00 (0.98 − 1.01)
Liver cirrhosis	63	262	1.14 (1.03 − 1.27)
COPD	70	911	1.17 (1.12 − 1.23)

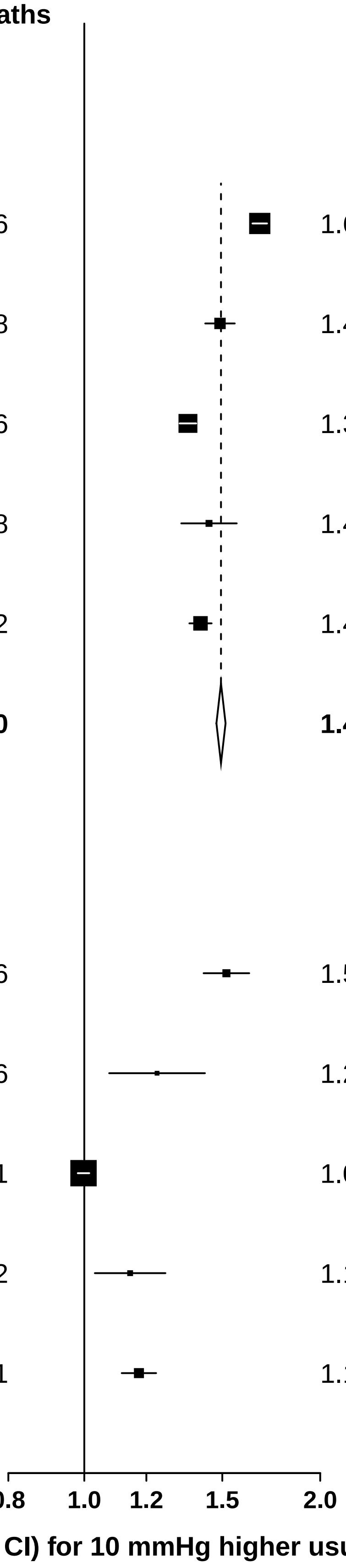

Hazard ratios (HR) at age 40−79 years, adjusted for age at risk (5−year groups), sex, region, education, smoking, alcohol and BMI. For each category, area of square is inversely proportional to the variance log hazard ratio, which also determines the confidence interval (CI). Exclusions as in Table 1; analyses of each non−vascular disease, further excluded participants with a prior diagnosis of the relevant disease at baseline. Deaths were identified from mortality registries, with the underlying cause of death obtained from the death certificate.

Figure S3: Vascular deaths attributable to elevated SBP in China, 2015

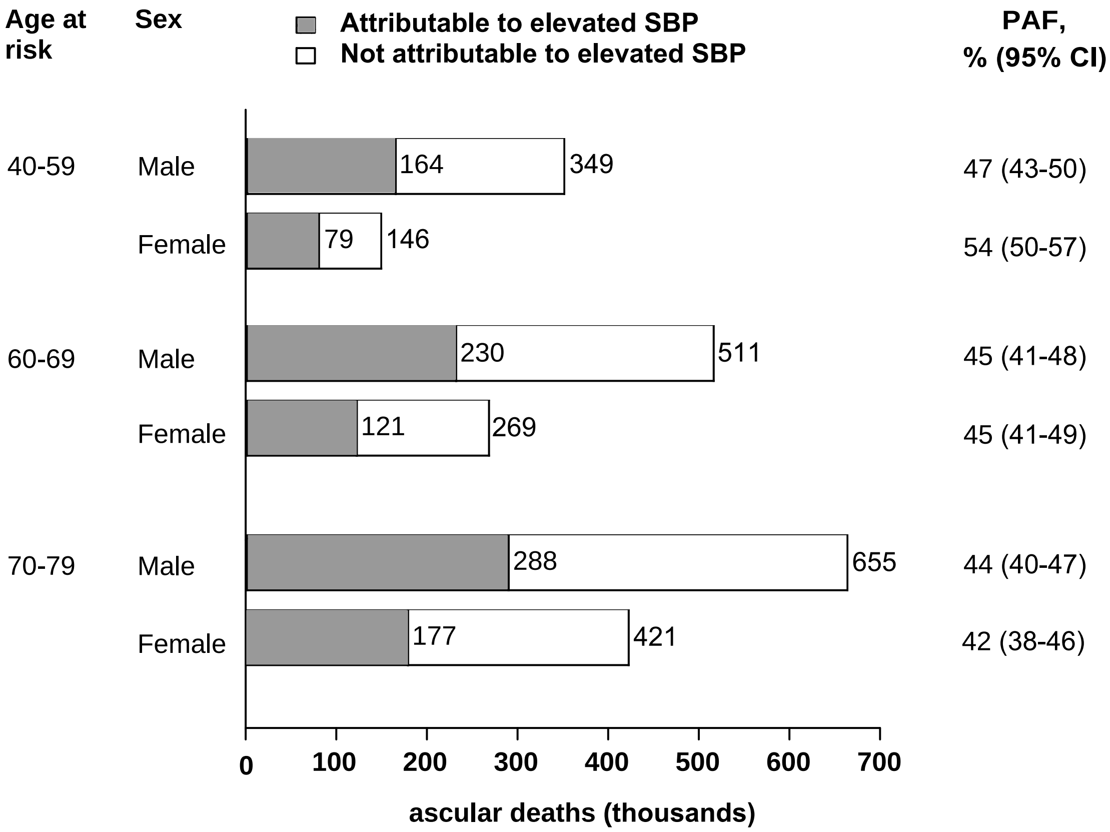

Population-attributable fractions (PAF) of elevated usual SBP (>120 mmHg) for vascular mortality were applied to the number of vascular deaths in China for 2015 (as estimated by the Global Burden of Disease Study 2015), to give the number of deaths attributable to elevate usual SBP for that year. At ages 40-79 years (both sexes combined), the overall PAF was 45 and the total number of deaths attributable to elevated SBP was 1.1 million.

Table S7: Incidence of vascular and non-vascular chronic disease: hazard ratios for 10 mm Hg higher usual SBP, progressively adjusted for potential confounders

	Mean age at event	Number of events	Hazard ratio (95% CI)		
			Model 1	Model 2	Model 3
**Major vascular disease**					
Ischaemic heart disease	66	4450	1.31(1.28-1.34)	1.31 (1.28-1.34)	1.30 (1.27-1.33)
Ischaemic stroke	64	29343	1.33 (1.31-1.34)	1.30 (1.29-1.31)	1.29 (1.28-1.31)
Intracerebral haemorrhage	64	6011	1.67 (1.65-1.70)	1.68 (1.65-1.71)	1.68 (1.65-1.70)
Other or unspecified stroke	63	1572	1.30 (1.26-1.35)	1.29 (1.24-1.34)	1.28 (1.23-1.33)
Other vascular	67	1286	1.32 (1.26-1.37)	1.34 (1.29-1.40)	1.33 (1.27-1.38)
All	64	42662	1.37 (1.36-1.38)	1.36 (1.35-1.37)	1.35 (1.34-1.36)
**Non-vascular disease**					
Chronic kidney disease	61	1940	1.41 (1.37-1.46)	1.40 (1.35-1.44)	1.35 (1.31-1.40)*
Diabetes	60	12241	1.26 (1.25-1.28)	1.14 (1.12-1.15)	1.14 (1.12-1.15)
Cancer	62	21044	0.99 (0.98-1.00)	0.99 (0.98-1.01)	0.99 (0.98-1.00)
Liver cirrhosis	59	1740	1.01 (0.97-1.05)	1.02 (0.98-1.06)	1.01 (0.97-1.06)
COPD	65	8478	0.96 (0.94-0.98)	0.98 (0.96-1.00)	0.98 (0.96-1.00)

Exclusions as in Table 1; analyses of each non-vascular disease, further excluded participants with a prior diagnosis of the relevant disease at baseline.
Model 1: Adjusted for age, sex and region;
Model 2: Adjusted for age, sex, region, education, smoking, alcohol and BMI;
Model 3: Adjusted for age, sex, region, education, smoking, alcohol , BMI, physical activity, diabetes, frequency of fruit consumption, and frequency of vegetable consumption.
*Not adjusting for diabetes: hazard ratio 1.39 (95% CI 1.34-1.43)

Table S8: Incidence of vascular and non-vascular chronic disease: hazard ratios for 10 mm Hg higher usual SBP in urban and rural areas

	Urban areas			Rural areas		
	Mean age at event	Number of events	Hazard ratio (95% CI)	Mean age at event	Number of events	Hazard ratio (95% CI)
**Major vascular disease**						
Ischaemic heart disease	66	1738	1.35 (1.30-1.40)	66	2712	1.28 (1.24-1.31)
Ischaemic stroke	65	15427	1.22 (1.21-1.24)	63	13916	1.37 (1.36-1.39)
Intracerebral haemorrhage	63	1409	1.71 (1.65-1.77)	64	4602	1.65 (1.62-1.68)
Other or unspecified stroke	64	662	1.35 (1.27-1.43)	62	910	1.25 (1.19-1.31)
Other vascular	69	605	1.34 (1.26-1.43)	68	681	1.32 (1.24-1.39)
All	65	19841	1.28 (1.26-1.29)	64	22821	1.41 (1.40-1.43)
**Non-vascular disease**						
Chronic kidney disease	63	813	1.42 (1.35-1.50)	60	1127	1.38 (1.32-1.44)
Diabetes	60	5140	1.18 (1.15-1.21)	59	7101	1.11 (1.09-1.14)
Cancer	62	9694	1.01 (0.99-1.03)	62	11350	0.98 (0.96-0.99)
Liver cirrhosis	60	689	1.04 (0.97-1.11)	59	1051	0.99 (0.94-1.05)
COPD	68	1763	0.95 (0.91-0.99)	64	6715	0.99 (0.97-1.01)

Hazard ratios are adjusted for age at risk (5-year groups), sex, area, education, smoking, alcohol and BMI. Exclusions as in Table 1; analyses of each non-vascular disease, further exclude participants with a prior diagnosis of the relevant disease at baseline.

Table S9: Incidence of vascular and non-vascular chronic disease: hazard ratios for 10 mm Hg higher usual SBP, excluding the first 3 years of follow-up and excluding those taking blood pressure-lowering medication at baseline

	Excluding the first 3 years of follow-up			Excluding participants taking blood-pressure lowering medication at baseline		
	Mean age at event	Number of events	Hazard ratio (95% CI)	Mean age at event	Number of events	Hazard ratio (95% CI)
**Major vascular disease**						
Ischaemic heart disease	67	2924	1.32 (1.29-1.36)	66	3992	1.31 (1.28-1.34)
Ischaemic stroke	64	20606	1.28 (1.27-1.29)	64	26673	1.30 (1.28-1.31)
Intracerebral haemorrhage	64	3579	1.69 (1.66-1.73)	63	5384	1.69 (1.66-1.72)
Other or unspecified stroke	63	1001	1.24 (1.18-1.30)	62	1429	1.29 (1.24-1.34)
Other vascular	69	856	1.35 (1.28-1.42)	67	1180	1.34 (1.28-1.40)
All	65	28966	1.34 (1.33-1.35)	64	38658	1.36 (1.35-1.37)
**Non-vascular disease**						
Chronic kidney disease	62	1335	1.41 (1.35-1.46)	61	1735	1.38 (1.33-1.43)
Diabetes	60	8576	1.14 (1.12-1.16)	60	11416	1.13 (1.11-1.15)
Cancer	63	12976	1.01 (0.99-1.02)	62	20065	0.99 (0.98-1.01)
Liver cirrhosis	60	1103	1.03 (0.97-1.08)	59	1684	1.03 (0.99-1.08)
COPD	66	5791	0.98 (0.96-1.00)	65	8066	0.97 (0.96-0.99)

Hazard ratios are adjusted for age at risk (5-year groups), sex, region, education, smoking, alcohol and BMI. Exclusions as in Table 1; analyses of each non-vascular disease, further excluded participants with a prior diagnosis of the relevant disease at baseline.

Table S10: Incidence of chronic kidney disease and diabetes versus usual SBP, by age at risk and sex

Age at risk, years	Chronic kidney disease			Diabetes		
	Mean age at event	No. of events	Hazard ratio (95% CI)	Mean age at event	No. of events	Hazard ratio (95% CI)
**Male**						
40-49	45	112	1.94 (1.70 - 2.21)	45	824	1.14 (1.06 - 1.22)
50-59	55	183	1.52 (1.36 - 1.70)	55	1436	1.07 (1.02 - 1.13)
60-69	64	258	1.37 (1.24 - 1.51)	64	1516	1.08 (1.04 - 1.13)
70-79	74	264	1.38 (1.25 - 1.52)	74	837	1.16 (1.10 - 1.24)
All	63	817	1.49 (1.41 - 1.57)	60	4613	1.10 (1.08 - 1.13)
**Female**						
40-49	45	194	1.46 (1.31 - 1.62)	46	1069	1.18 (1.12 - 1.24)
50-59	55	350	1.32 (1.22 - 1.41)	55	2686	1.14 (1.11 - 1.17)
60-69	64	338	1.41 (1.31 - 1.51)	64	2602	1.17 (1.14 - 1.20)
70-79	74	241	1.21 (1.10 - 1.33)	74	1271	1.11 (1.07 - 1.16)
All	60	1123	1.35 (1.29 - 1.40)	60	7628	1.15 (1.13 - 1.17)
**Overall**	61	1940	1.40 (1.35-1.44)	60	12241	1.14 (1.12-1.15)

Hazard ratios are adjusted for age at risk (5-year groups), sex (where appropriate), region, education, smoking, alcohol and BMI. Exclusions as in Table 1; analyses of each non-vascular disease, further excluded participants with a prior diagnosis of the relevant disease at baseline.

Table S11: Incidence of chronic kidney disease and cancer versus usual SBP, by disease subtype

	Mean age at event	No. of events	Hazard ratio (95% CI)
**Chronic kidney disease, by subtype***			
Hypertensive renal disease	60	241	1.43 (1.31-1.57)
Renal complications from diabetes	63	529	1.34 (1.26-1.43)
Other/unspecified chronic kidney disease	61	1170	1.43 (1.37-1.49)
All	61	1940	1.40 (1.35-1.44)
**Cancer, by site†**			
Lung	65	3722	0.99 (0.97-1.02)
Colorectal	63	2102	0.98 (0.95-1.02)
Liver	62	1925	0.99 (0.96-1.03)
Oesophagus	64	1701	1.01 (0.97-1.05)
Other/unspecified cancer	61	11594	1.00 (0.99-1.02)
All	62	21044	0.99 (0.98-1.01)

Hazard ratios are adjusted for age at risk (5-year groups), sex, regioin, education, smoking, alcohol and BMI. Exclusions as in Table 1; analyses of each non-vascular disease, further excluded participants with a prior diagnosis of the relevant disease at baseline.

* ICD 10 codes for chronic kidney disease: hypertensive renal disease (I12, I13); renal complications from diabetes (E10.2, E11.2, E12.2, E13.2, E14.2); and other chronic kidney disease (N03, N18, N26, N27)

† ICD 10 codes for cancer: Oesophagus (C15); Colorectal (C18-C21); Liver (C22); Lung (C33-C34); other cancer (remainder of chapter C00-C97).

Figure S4: Incidence of ischaemic heart disease, ischaemic stroke and intracerebral haemorrhage versus usual DBP

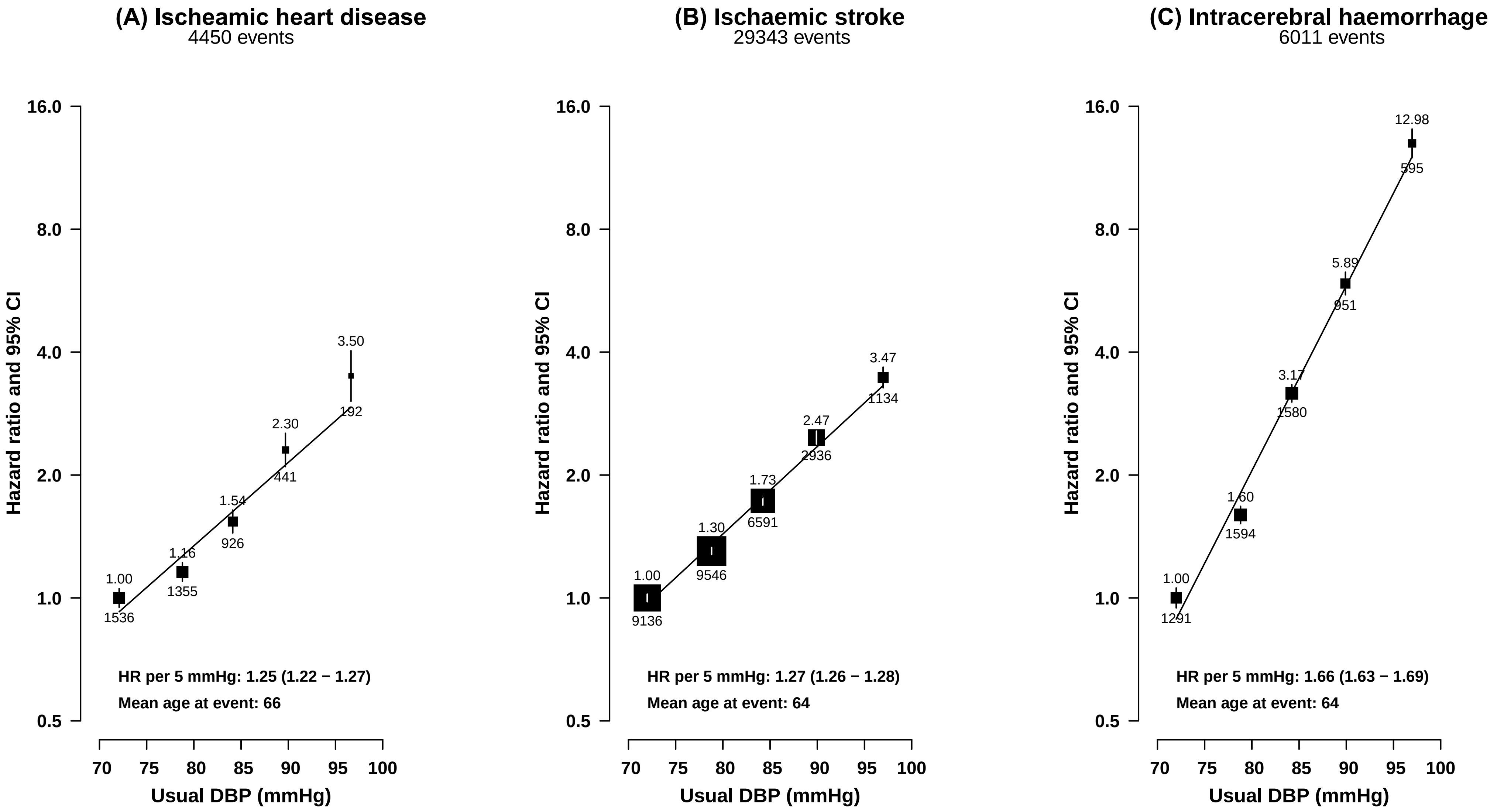

Hazard ratios (HR) at age 40-79 years, adjusted for age at risk (5-year groups), sex, region, education, smoking, alcohol and BMI. For each category, the area of each square is inversely proportional to the variance of the category-specific log risk, which also determines the confidence interval (CI). HR is shown above each square and numbers of events below. Exclusions as in Table 1.

Table S12: Incidence of ischaemic heart disease, ischaemic stroke and intracerebral haemorrhage versus usual DBP, by age at risk and sex

Age at risk, years	Ischaemic heart disease			Ischaemic stroke			Intracerebral haemorrhage		
	Mean age at event	No. of events	Hazard ratio (95% CI)	Mean age at event	No. of events	Hazard ratio (95% CI)	Mean age at event	No. of events	Hazard ratio (95% CI)
**Male**									
40-49	46	192	1.43 (1.31-1.57)	46	941	1.49 (1.43-1.55)	45	297	1.88 (1.76-2.00)
50-59	55	487	1.35 (1.27-1.44)	55	3058	1.42 (1.38-1.46)	55	667	1.89 (1.80-1.98)
60-69	65	774	1.23 (1.16-1.29)	65	4475	1.33 (1.30-1.36)	65	1107	1.68 (1.61-1.74)
70-79	74	1046	1.16 (1.11-1.22)	74	4757	1.16 (1.14-1.19)	74	1124	1.44 (1.38-1.51)
All	65	2499	1.25 (1.21-1.28)	64	13231	1.31 (1.29-1.33)	64	3195	1.67 (1.63-1.71)
**Female**									
40-49	46	98	1.50 (1.32-1.70)	46	1131	1.44 (1.38-1.49)	46	317	1.90 (1.79-2.01)
50-59	55	303	1.36 (1.25-1.47)	55	4277	1.27 (1.24-1.30)	55	736	1.82 (1.74-1.90)
60-69	65	609	1.23 (1.16-1.31)	64	5841	1.22 (1.19-1.24)	64	889	1.64 (1.57-1.71)
70-79	75	941	1.18 (1.12-1.24)	74	4863	1.15 (1.12-1.17)	74	874	1.33 (1.27-1.40)
All	67	1951	1.25 (1.21-1.29)	64	16112	1.23 (1.22-1.25)	63	2816	1.65 (1.61-1.69)
**Overall**	66	4450	1.25 (1.22-1.27)	64	29343	1.27 (1.26-1.28)	64	6011	1.66 (1.63-1.69)

Hazard ratios are adjusted for age at risk (5-year groups), sex (where appropriate), region, education, smoking, alcohol and BMI. Exclusions as in Table 1.

Figure S5: Incidence of vascular and non−vascular chronic disease: hazard ratio for 5 mmHg higher usual DBP

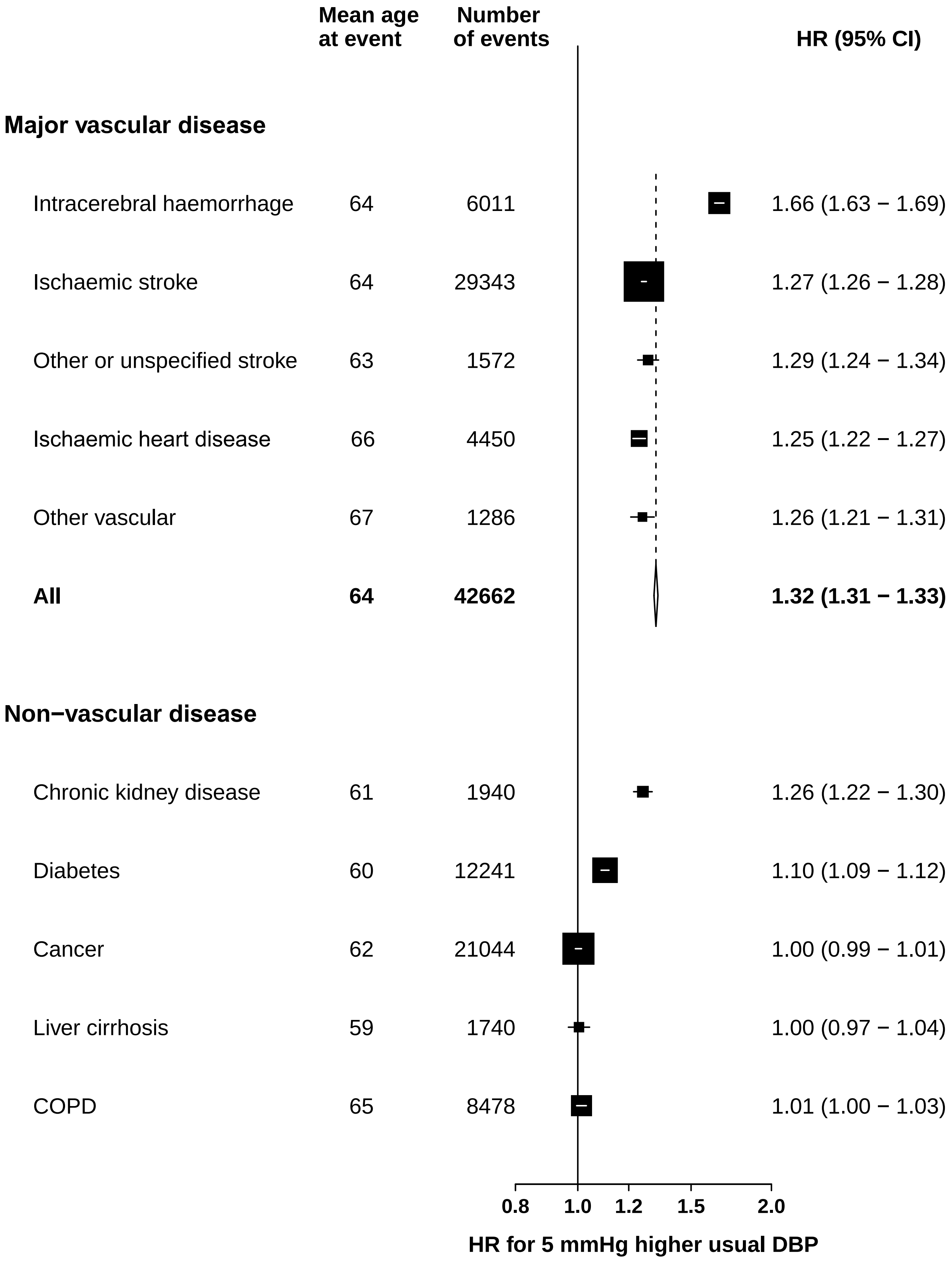

Hazard ratios (HR) at age 40−79 years, adjusted for age at risk (5−year groups), sex, region, education, smoking, alcohol and BMI. For each category, area of square is inversely proportional to the variance log hazard ratio, which also determines the confidence interval (CI). Exclusions as in Table 1; analyses of each non-vascular disease, further excluded participants with a prior diagnosis of the relevant disease at baseline.

Figure S6: Incidence of vascular and non−vascular chronic disease versus usual DBP

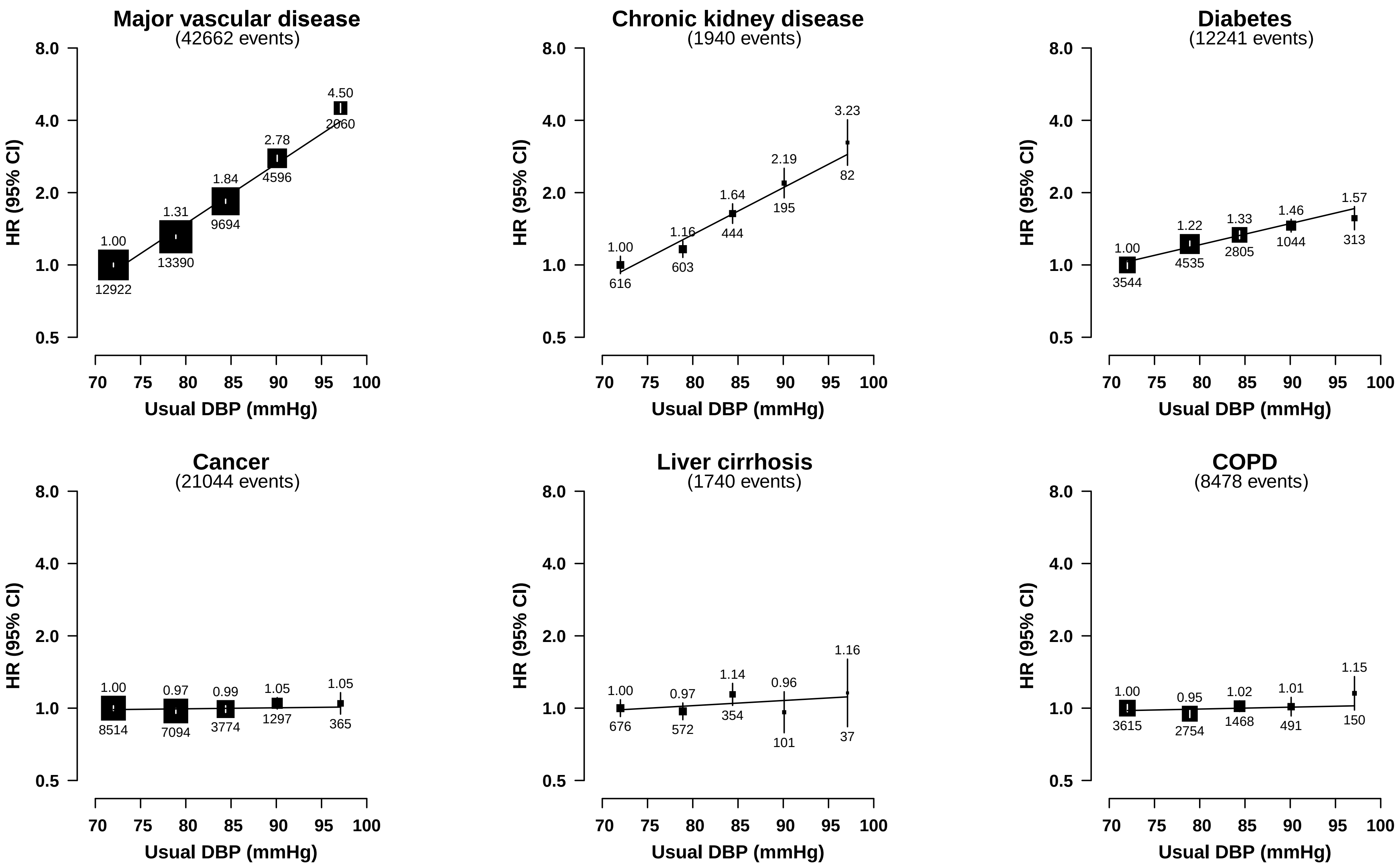

Hazard ratios (HR) at age 40−79 years, adjusted for age at risk (5−year groups), sex, region, education, smoking, alcohol and BMI. For each category, the area of each square is inversely proportional to the variance of the category−specific log risk, which also determines the confidence interval (CI). HR is shown above each square and numbers of events below. Exclusions as in Table 1; analyses of each non-vascular disease, further excluded participants with a prior diagnosis of the relevant disease at baseline.

China Kadoorie Biobank collaborative group and members

International Steering Committee: Junshi Chen, Zhengming Chen (PI), Robert Clarke, Rory Collins, Yu Guo, Liming Li (PI), Jun Lv, Richard Peto, Robin Walters.

International Co-ordinating Centre, Oxford: Daniel Avery, Derrick Bennett, Ruth Boxall, Fiona Bragg, Yumei Chang, Yiping Chen, Zhengming Chen, Robert Clarke, Huaidong Du, Simon Gilbert, Alex Hacker, Michael Holmes, Andri Iona, Christiana Kartsonaki, Rene Kerosi, Om Kurmi, Sarah Lewington, Garry Lancaster, Kuang Lin, John McDonnell, Iona Millwood, Qunhua Nie, Jayakrishnan Radhakrishnan, Paul Ryder, Sam Sansome, Dan Schmidt, Paul Sherliker, Rajani Sohoni, Becky Stevens, Iain Turnbull, Robin Walters, Jenny Wang, Lin Wang, Neil Wright, Ling Yang, Xiaoming Yang.

National Co-ordinating Centre, Beijing: Zheng Bian, Ge Chen, Yu Guo, Xiao Han, Can Hou, Jun Lv, Pei Pei, Shuzhen Qu, Yunlong Tan, Canqing Yu.

10 Regional Co-ordinating Centres: Qingdao Qingdao CDC: Zengchang Pang, Ruqin Gao, Shaojie Wang, Yongmei Liu, Ranran Du, Yajing Zang, Liang Cheng, Xiaocao Tian, Hua Zhang. Licang CDC: Silu Lv, Junzheng Wang, Wei Hou. **Heilongjiang** Provincial CDC: Jiyuan Yin, Ge Jiang, Xue Zhou. Nangang CDC: Liqiu Yang, Hui He, Bo Yu, Yanjie Li, Huaiyi Mu, Qinai Xu, Meiling Dou, Jiaojiao Ren. **Hainan** Provincial CDC: Shanqing Wang, Ximin Hu, Hongmei Wang, Jinyan Chen, Yan Fu, Zhenwang Fu, Xiaohuan Wang. Meilan CDC: Min Weng, Xiangyang Zheng, Yilei Li, Huimei Li,Yanjun Wang. **Jiangsu** Provincial CDC: Ming Wu, Jinyi Zhou, Ran Tao, Jie Yang. Suzhou CDC: Chuanming Ni, Jun Zhang, Yihe Hu, Yan Lu, , Liangcai Ma, Aiyu Tang, Shuo Zhang, Jianrong Jin, Jingchao Liu. **Guangxi** Provincial CDC: Zhenzhu Tang, Naying Chen, Ying Huang. Liuzhou CDC: Mingqiang Li, Jinhuai Meng, Rong Pan, Qilian Jiang, Weiyuan Zhang, Yun Liu, Liuping Wei, Liyuan Zhou, Ningyu Chen, Hairong Guan. **Sichuan** Provincial CDC: Xianping Wu, Ningmei Zhang, Xiaofang Chen, Xuefeng Tang. Pengzhou CDC: Guojin Luo, Jianguo Li, Xiaofang Chen, Xunfu Zhong, Jiaqiu Liu, Qiang Sun. **Gansu** Provincial CDC: Pengfei Ge, Xiaolan Ren, Caixia Dong. Maiji CDC: Hui Zhang, Enke Mao, Xiaoping Wang, Tao Wang, Xi Zhang. **Henan** Provincial CDC: Ding Zhang, Gang Zhou, Shixian Feng, Liang Chang, Lei Fan. Huixian CDC: Yulian Gao, Tianyou He, Huarong Sun, Pan He, Chen Hu, Qiannan Lv, Xukui Zhang. **Zhejiang** Provincial CDC: Min Yu, Ruying Hu, Hao Wang. Tongxiang CDC: Yijian Qian, Chunmei Wang, Kaixue Xie, Lingli Chen, Yidan Zhang, Dongxia Pan. **Hunan** Provincial CDC: Yuelong Huang, Biyun Chen, Li Yin, Donghui Jin, Huilin Liu, Zhongxi Fu, Qiaohua Xu. Liuyang CDC: Xin Xu, Hao Zhang, Youping Xiong, Huajun Long, Xianzhi Li, Libo Zhang, Zhe Qiu.
